# Assessment of AI-Driven Large Language Models for Orthodontic Aesthetic Scoring Using the IOTN-AC

**DOI:** 10.3390/diagnostics15233048

**Published:** 2025-11-29

**Authors:** Ahmet Yıldırım, Orhan Cicek

**Affiliations:** Department of Orthodontics, Faculty of Dentistry, Zonguldak Bulent Ecevit University, Zonguldak 67600, Türkiye; orhancicek@beun.edu.tr

**Keywords:** orthodontic treatment need, occlusal indices, Index of Orthodontic Treatment Need, aesthetic component, artificial intelligence, large language model

## Abstract

**Background/Objectives**: The aim of this study was to evaluate the accuracy of aesthetic assessments performed by artificial intelligence (AI)-based large language models (LLMs) using the Aesthetic Component of the Index of Orthodontic Treatment Need (IOTN-AC), which is widely applied to determine the need for orthodontic treatment. **Methods**: A total of 150 frontal intraoral photographs from patients in the permanent dentition, scored from 1 to 10 on the IOTN-AC, were assessed by two AI-based LLMs (ChatGPT-5 and ChatGPT-5 Pro). Two experienced clinicians independently scored all photographs, with one evaluator’s scores used as the reference (κ = 0.91, ICC = 0.88). Model performance was analyzed by comparing IOTN-AC scores and treatment need classifications. In addition, performance parameters such as accuracy, precision, specificity, and sensitivity were evaluated. Statistical analyses included Spearman correlation, Cohen’s Kappa, ICC, Mean Absolute Error (MAE), Wilcoxon signed-rank test, and Bland–Altman analysis. **Results**: Both models demonstrated positive and significant correlations with the reference values for scoring and classification (*p* < 0.001). Compared to GPT-5 Pro, the GPT-5 model exhibited superior performance, with a lower error rate (MAE = 1.47) and higher classification accuracy (66.7%). Bland–Altman analysis showed that most predictions fell within the 99% confidence interval, and regression analysis revealed no systematic bias (*p* > 0.05). Conversely, the models failed to achieve consistently high performance in each of the performance parameters. **Conclusions**: The findings revealed that although AI-based LLMs are promising, statistical accuracy alone is insufficient for safe clinical use, and they should demonstrate consistently high performance across all parameters.

## 1. Introduction

In orthodontics, various indices have been developed to objectively evaluate occlusal relationships, some of which determine access to publicly funded orthodontic treatment [1]. The main types include diagnostic classification [2,3], epidemiological [4,5], treatment complexity [2], treatment outcome [2], and treatment need indices [6,7,8]. The Index of Orthodontic Treatment Need (IOTN), an adaptation of treatment need indices, identifies individuals likely to benefit from orthodontic treatment for oral health and aesthetics. It comprises two components: the Dental Health Component (DHC), based on clinical anomalies, and the Aesthetic Component (AC), which uses a standardized photographic scale from 1 (most aesthetic) to 10 (least aesthetic) to assess aesthetic impairment [9,10].

Conventional AC assessments take 1–3 min when performed by experienced clinicians [2]. Artificial intelligence (AI) tools, particularly large language models (LLMs), have been proposed to speed up this process by integrating visual and linguistic data for more comprehensive analyses [11]. LLMs, such as ChatGPT (OpenAI, San Francisco, CA, USA), can simulate human-like interactions, generate coherent text, answer questions, perform visual analyses, and support various language-related tasks [12,13,14].

The ability of LLMs to process not only textual data, but also visual information [11], makes it worthwhile to investigate the extent to which the Aesthetic Component of the Index of Orthodontic Treatment Need (IOTN-AC), one of the most commonly used aesthetic assessment scales in orthodontics, can be accurately classified through these models.

IOTN-AC evaluations performed by LLMs could provide clinicians with more objective and reproducible results, independent of subjective judgments. They may also reduce assessment time, improve efficiency, and enable patients to assess their own aesthetic perceptions in a digital environment. Beyond these practical benefits, the integration of AI-based LLM assessments into orthodontic practice could contribute to more consistent treatment planning and enhance communication between clinicians and patients. Such developments hold the potential to support evidence-based decision-making and improve patient satisfaction in clinical settings. Considering these potential contributions, it is clear that the reliability and clinical validity of IOTN-AC assessments generated by AI-based LLMs should be evaluated.

Therefore, this study aimed to examine the reliability and accuracy of aesthetic assessments performed by LLMs using the IOTN-AC and to assess their agreement with clinical evaluators.

## 2. Materials and Methods

### 2.1. Ethical Approval and Study Sample

For this study, ethical approval was obtained from the Non-Interventional Clinical Research Ethics Committee of Zonguldak Bülent Ecevit University (approval protocol no: 2025/15-29, date: 3 September 2025). A total of 150 frontal intraoral photographs of patients who had applied for orthodontic treatment at the Department of Orthodontics, Zonguldak Bülent Ecevit University, were used. The photographs were organized into 10 groups, each consisting of 15 images representing every score from 1 to 10 on the IOTN-AC index. The groups were formed with attention to maintaining a homogeneous distribution of male and female patients in the permanent dentition period.

### 2.2. Evaluation Procedure of AI-Based LLMs

Since GPT-based platforms are among the most frequently used AI systems for informational purposes in healthcare, the GPT-5 and GPT-5 Pro models were selected for evaluation in this study [15]. Accordingly, the 150 photographs were randomly presented to the GPT-5 and GPT-5 Pro platforms with the command: “Classify this frontal intraoral photograph within the scope of the Aesthetic Component (AC) of the Index of Orthodontic Treatment Need (IOTN) according to the criteria ‘AC 1–4: no treatment need; AC 5–7: borderline need; AC 8–10: definite treatment need’ and score it between 1 and 10.” After each photograph assessment, the session was reset and a new window was opened before uploading the next photograph. All commands were submitted using the same laptop (MacBook Air M3, 16 GB RAM; Apple, Cupertino, CA, USA) with a 4.5G internet connection and a VPN server (version 3.9; Astrill Systems Corp, Santa Clara, CA, USA). Each radiograph and command was submitted to each LLM only once, without additional prompts or rephrasing if the model failed to respond. No domain-specific pretraining or additional textual information related to the IOTN-AC scale was provided to the models. They were evaluated entirely in their existing publicly available forms, with the aim of reflecting real-world clinical usage scenarios. To assess temporal consistency, a randomly selected subsample representing ten percent of the question set was resubmitted to the models one week later. The Cohen’s Kappa and Intraclass Correlation Coefficient (ICC) values calculated from this subsample were found to be high, with Kappa = 0.80 and ICC = 0.84.

### 2.3. Reference Evaluations by Clinicians

In this study, a two-step comparative approach was adopted to analyze the ability of LLMs to perform evaluations within the scope of the IOTN-AC. Two experienced clinicians (A.Y., O.C.) independently classified the same set of frontal intraoral photographs according to treatment need based on the IOTN-AC (AC 1–4: no treatment need, AC 5–7: borderline need, AC 8–10: definite treatment need) and scored them on the IOTN-AC scale from 1 to 10, carrying out all assessments in a fully blinded manner without any access to the LLM-generated scores and without any communication with each other. Cohen’s kappa (0.91) and the ICC (0.88) confirmed high inter-evaluator consistency, after which the scores of evaluator A.Y. were designated as the reference for comparing the AI models’ predictions.

### 2.4. Data Analysis and Performance Metrics

The analysis was performed in three stages:

1. Comparison of the LLMs’ classification of each photograph with the reference classifications.
2. Evaluation of the agreement between the AC scores (1–10) assigned by the LLMs and the reference scores.
3. Assessment of the models’ classification performance using accuracy, sensitivity, precision, and specificity [11].

### 2.5. Statistical Analysis

The obtained data were statistically analyzed using the Statistical Package for the Social Sciences (SPSS, version 26; IBM Corporation, New York, NY, USA). The normality of the data distribution was assessed using the Kolmogorov–Smirnov test. To evaluate the agreement between the IOTN-AC scores of the LLMs and the reference scores, the Spearman Correlation Coefficient was used. To assess the differences between the LLM scores and the reference scores, the Mean Absolute Error (MAE) was calculated, and the Wilcoxon signed-rank test was applied to examine the differences between the mean MAE values of the two LLMs.

In addition, Bland–Altman analysis was performed to identify potential systematic bias between the LLMs and the reference IOTN-AC scores. Scatter plot graphs were used to visually examine the distributional agreement between the predictions of the LLMs and the reference scores, as well as to evaluate the score ranges in which the models were concentrated and the regions where deviations were more pronounced. Furthermore, to assess the classification performance of the models, Cohen’s Kappa analysis was conducted separately for each class. A *p*-value of less than 0.05 was considered statistically significant.

## 3. Results

The analyses showed that both models had a statistically significant agreement with the reference data, with GPT-5 Pro model yielding a Cohen’s Kappa value of 0.412, indicating moderate agreement, and the GPT-5 model yielding a Cohen’s Kappa value of 0.507, indicating moderate-to-good agreement (both *p* < 0.001). In addition, the GPT-5 Pro model correctly classified the ‘‘No need” group at a significant rate of 60%. The GPT-5 model correctly classified the “No need” group at a significant rate of 58.3% (see Table 1).

The performance parameters related to the classification abilities of the models are presented in Table 2.

GPT-5 achieved a higher overall accuracy across all categories, reaching 66.7%. According to the results of the Spearman correlation analysis, both the GPT-5 and GPT-5 Pro models demonstrated statistically significant positive correlations between their IOTN-AC scores and the reference scores (*p* < 0.001). In addition, the models also showed high correlations with each other, reflecting similar classification tendencies (*p* < 0.001) (see Table 3).

When the MAE values calculated against the reference scores were examined, the mean MAE was found to be 1.68 ± 1.34 (Median = 1.00) for GPT-5 Pro and 1.47 ± 1.29 (Median = 1.00) for GPT-5. The Wilcoxon signed-rank test showed that the GPT-5 model produced statistically significantly lower errors compared to the GPT-5 Pro model, providing results closer to the reference scores (Z = −2.32, *p* = 0.020). Table 4 provides a summary of the performance results obtained from the LLMs in the context of IOTN-AC classification.

**Figure 1:**
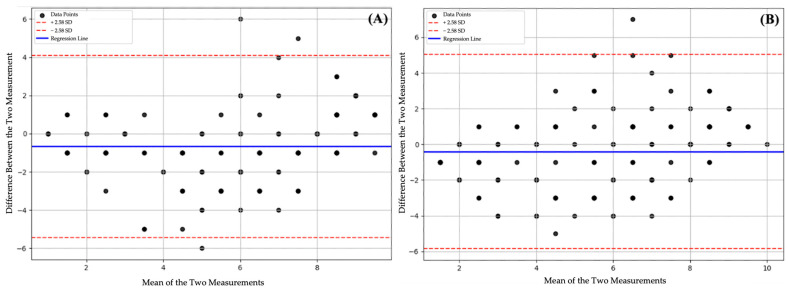
Bland–Altman plots illustrating the agreement between the IOTN-AC score predictions and the reference data. (**A**) GPT-5 model. (**B**) GPT-5 Pro model. The red dashed lines represent the 99% confidence limits (±2.58 SD). The slope of the blue regression line is not statistically significant (*p* > 0.05).

In the Bland–Altman analyses for GPT-5 Pro and GPT-5, it was observed that the majority of the differences between the models’ predictions of IOTN-AC scores and the reference evaluations fell within the 99% confidence interval (±2.58 SD). Regression analysis indicated no significant trend for either GPT-5 Pro or GPT-5, with lines remaining horizontal (*p* > 0.05) (see Figure 1).

**Figure 2:**
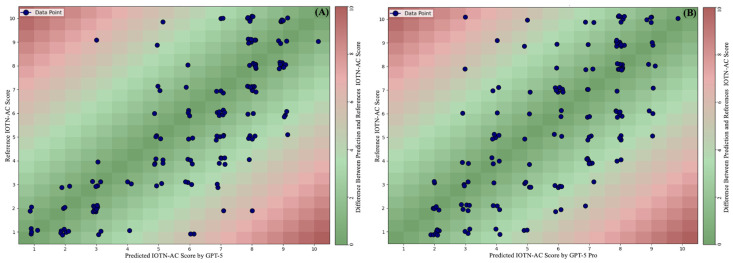
Scatter plots illustrating the agreement between the IOTN-AC score predictions and the reference data. (**A**) GPT-5 model. (**B**) GPT-5 Pro model. Each blue dot represents a model prediction. The background colors indicate the absolute difference between the model prediction and the reference score, with dark green areas representing the most accurate predictions and red areas representing the highest errors.

The scatter plots presented in Figure 2 provide a comparative illustration of the agreement between the IOTN-AC score predictions of the GPT-5 Pro and GPT-5 models and the reference values. It can be observed that the data points of both models are concentrated in areas close to the reference scores.

## 4. Discussion

General dentists play a key role in ensuring that patients in need of orthodontic treatment have access to appropriate healthcare services. They are expected to monitor dental development, detect deviations early, and provide appropriate referrals [1]. The literature reports that general dentists often have difficulty assessing malocclusions and making referrals. Additional training is needed to use the IOTN [16,17,18]. An objective evaluation of the AC of the index requires calibration, which can only be achieved through specialized training [19]. Errors in this area may lead to delays in treatment timing.

Automating the IOTN-AC evaluation could reduce diagnostic inconsistencies and alleviate the clinical workload. Furthermore, intraoral images obtained with mobile devices could enable patients to perform preliminary assessments [20]. Although a mobile application developed to facilitate IOTN assessment has shown a certain level of success, requiring installation on devices limits accessibility [1,21]. Directing evaluation support for the AC of the IOTN to web-based AI platforms eliminates the need for installation. However, analyzing the reliability of such methods is critical. In this regard, our study examined the performance of AI–based LLMs in IOTN-AC classification.

Recent studies have demonstrated the applicability of LLMs in areas such as risk assessment, diagnosis, anomaly detection, appointment scheduling, and scientific research [22]. Within this scope, LLMs have been evaluated across various clinical domains, including restorative treatment planning [23], oral lesion identification [24], implantology support [25], and endodontics [26].

In light of previous studies in the literature in which LLMs have been frequently evaluated across various clinical domains, the present research likewise assessed the performance of LLMs in the classification of the IOTN-AC. According to the results, the scorings performed by the two AI-based LLMs for the IOTN-AC showed statistically significant correlations with the reference scores (Spearman’s rho = 0.772 for GPT-5 and 0.685 for GPT-5 Pro, *p* < 0.001 for both). Compared to a previous study reporting a 0.88 correlation between two orthodontic treatment need indices [27], the AI systems did not reach the desired level but performed above random. Based on the Bland–Altman analyses, the absence of a significant slope in the regression line indicates that no systematic bias was present and that the prediction errors of the models were independent of the score levels. However, the wide limits of agreement reduce the reliability of the models’ classification performance. On the other hand, the clustering of predictions around the central areas of the reference values in the scatter plots points to a structural consistency and suggests that these models have a foundation that can be further improved.

The two different LLMs produced varying levels of success in diagnostic performance measures such as sensitivity, specificity, and accuracy based on the IOTN-AC. Although the LLMs outperformed the success rates of previously developed AI–based machine learning systems for IOTN-based classification in certain aspects [28], these results should be interpreted with caution. High performance across all parameters is required for clinical reliability. Indeed, studies analyzing the performance of the index have shown that sensitivity and specificity values were simultaneously high and that strong consistency was achieved across all categories [29,30].

Previous studies have shown that the performance of CNN-based systems developed for IOTN assessment varies considerably. While some investigations reported consistent yet clinically limited accuracy, others demonstrated substantially higher levels of diagnostic precision and reliability. Sabri et al. [28] aimed to classify malocclusion based on the IOTN using a combination of convolutional neural networks (CNN) and knowledge-based systems (KBS), and reported an accuracy of 61.20%, with precision and recall values of 68% and 61%, respectively. Talaat et al. [31] investigated the validity of a CNN-based artificial intelligence application developed to assess orthodontic treatment need from clinical intraoral images and reported that the model was able to detect and localize malocclusions with an accuracy of 99.99%, a precision of 99.79%, and a recall of 100%.

Based on the study’s results, both models achieved higher accuracy rates in the “Definite need” category than in other classifications when their class-based performances were evaluated. This finding suggests that individuals with obvious aesthetic issues were more easily identified by the LLMs. The GPT-5 model’s high precision value of 97.2% in the “No need” group demonstrates that nearly all individuals classified as “No need” by the model truly did not require treatment. Furthermore, its specificity value of 99% indicates that the model was highly effective in avoiding the misclassification of individuals who actually required treatment as “No need.”

These parameters suggest that the risk of leaving a patient who requires treatment untreated is low, which is reassuring in terms of protecting patient rights. However, the sensitivity rate remaining at only 58% indicates that a large proportion of individuals who did not actually require treatment were incorrectly classified as if they were in need of treatment. This outcome poses a risk in terms of preventing unnecessary treatments, ensuring the efficient use of resources, and reducing the overall system burden. Moreover, this low sensitivity may also lead to unnecessary anxiety for patients. On the other hand, the high sensitivity of 86.7% achieved by GPT-5 in the “Definite need” group shows that the vast majority of individuals with a definite need for treatment were correctly identified. This is highly important to ensure that patients with a clear treatment need are not left untreated. However, the precision rate of 65.0% indicates that the model also classified individuals without a definite treatment need into this group, which may create increased demand and potentially delay access to treatment for those who genuinely require it. In general, the GPT-5 Pro model also demonstrated similar parameter performances, albeit with lower percentages.

Depending on the perspective of the evaluating party, the results obtained can be interpreted differently. For institutions financing the service, preventing unnecessary expenditures is a priority; therefore, erroneous treatment recommendations are undesirable. However, from the perspective of patients or healthcare providers, the main concern is ensuring that individuals in need of treatment are not excluded and can receive necessary care. For this reason, the primary priority in systems financed by public resources should be ensuring access to treatment for all individuals who truly require it. Financing certain cases with lower treatment needs should be considered less problematic than excluding an eligible individual. Furthermore, evaluations should be impartial [32] and not solely based on statistical success rates. Each performance indicator should prioritize patient benefit and protect the right to treatment access.

The fact that the data in this study were obtained from a single center may limit demographic and clinical diversity, thereby reducing the generalizability of the findings to broader populations. In addition, the limited sample size may constrain the statistical power of the results and the precision of inferences regarding model performance. Therefore, further research with larger sample sizes and multicenter datasets is warranted. On the other hand, although the use of reference scores generated with a high level of agreement among experienced orthodontists enabled a systematic comparison of the two LLMs, these assessments are inherently subjective. Evaluations performed by different experts could yield varying outcomes, potentially altering the overall findings. Additionally, only two models were evaluated in this study, and the investigation of other models with different architectures and visual-processing capabilities is recommended for future research.

## 5. Conclusions

Based on the findings of the study, the following conclusions were reached:

• Although AI-based LLMs showed statistically significant and positive correlations with the reference IOTN-AC assessments, their current agreement levels remain insufficient for reliable clinical use.
• The models exhibited variable performance across key evaluation metrics, indicating that consistent and high-level accuracy, sensitivity, specificity, and precision have not yet been achieved.
• Despite these limitations, the results demonstrate that LLMs possess notable potential. With targeted domain-specific fine-tuning and improved training strategies, these models may become valuable supportive tools in future orthodontic assessment workflows.

## Figures and Tables

**Table 1:** Statistical results of AI-based LLMs for IOTN-AC classification according to reference data.

LLMs	IOTN-AC	References				*Cohen’s Kappa* (%95–CI)	*p*
		No Need	Borderline Need	Definite Need	Total		
		*n* (%)	*n* (%)	*n* (%)	*n* (%)		
GPT-5 Pro	No need	36 (60) ^a^	22 (36.7) ^b^	2 (3.3) ^c^	60 (100)	0.412 (0.292–0.517)	<0.001 *
	Borderline need	8 (17.8) ^a^	22 (48.9) ^b^	15 (33.3) ^a,b^	45 (100)		
	Definite need	3 (6.7) ^a^	9 (20) ^a^	33 (73.3) ^b^	45 (100)		
GPT-5	No need	35 (58.3) ^a^	23 (38.3) ^b^	2 (3.3) ^c^	60 (100)	0.507 (0.396– 0.609)	<0.001 *
	Borderline need	0 (0) ^a^	26 (57.8) ^b^	19 (42.2) ^b^	45 (100)		
	Definite need	1 (2.2) ^a^	5 (11.1) ^a^	39 (86.7) ^b^	45 (100)		

*n*: sample, %: percentage, CI: Confidence Interval, *p*: significance level, *: *p* < 0.05, ^a,b,c^: There is a statistically significant difference between groups with different top index letters in the same row.

**Table 2:** Comparison of performance metrics for AI-based LLMs.

LLMs	Treatment Need	Sensitivity	Specificity	Precision	Accuracy
GPT-5 Pro	No need	60.0	87.8	76.6	60.7
	Borderline need	48.9	70.5	41.5	
	Definite need	73.3	83.8	66.0	
GPT-5	No need	58.3	98.9	97.2	66.7
	Borderline need	57.8	73.3	48.1	
	Definite need	86.7	80.0	65.0	

All values presented in the table are expressed as percentages (%).

**Table 3:** Correlation analysis between reference data and LLMs in IOTN-AC scores.

Groups		References	GPT-5 Pro	GPT-5
References	Spearman’s rho	1	0.685	0.772
	*p*		<0.001 *	<0.001 *
GPT-5 Pro	Spearman’s rho		1	0.868
	*p*			<0.001 *
GPT-5	Spearman’s rho			1
	*p*			

*p*: significance level, *: *p* < 0.05.

**Table 4:** Summary of the performance results of LLMs in IOTN-AC classification.

LLMs	*Cohen’s Kappa* (*Classification Agreement*)	Spearman’s rho (*Correlation of Scores*)	MAE *Mean ± SD* (*Median*)	Accuracy (%)
GPT-5 Pro	0.412	0.685	1.68 ± 1.34 (1.00)	60.7
GPT-5	0.507	0.772	1.47 ± 1.29 (1.00)	66.7

MAE: Mean Absolute Error, SD: Standard Deviation.

## Data Availability

Data are entirely contained within the article.
